# Supplementary material for: Strain differences between C57Bl/6 and DBA/2 mice (Mus musculus) in delayed matching and nonmatching-to-position tasks: impact of sample responses and delay intervals
Source: PeerJ. 2025 Mar 24;13:e19200. doi: 10.7717/peerj.19200 (PMC11949106; doi:10.7717/peerj.19200)

Strain Differences Between C57Bl/6 and DBA/2 Mice (Mus musculus) in Delayed Matching and Nonmatching-to-Position Tasks: Impact of Sample Responses and Delay Intervals

Kazuhiro Goto

Supplementary materials

Fig 1S. Individual data from DNMTP test with variable delay intervals. The horizontal axis represents the ratio of target to distractor luminance. Group data are shown in figure 3(B).

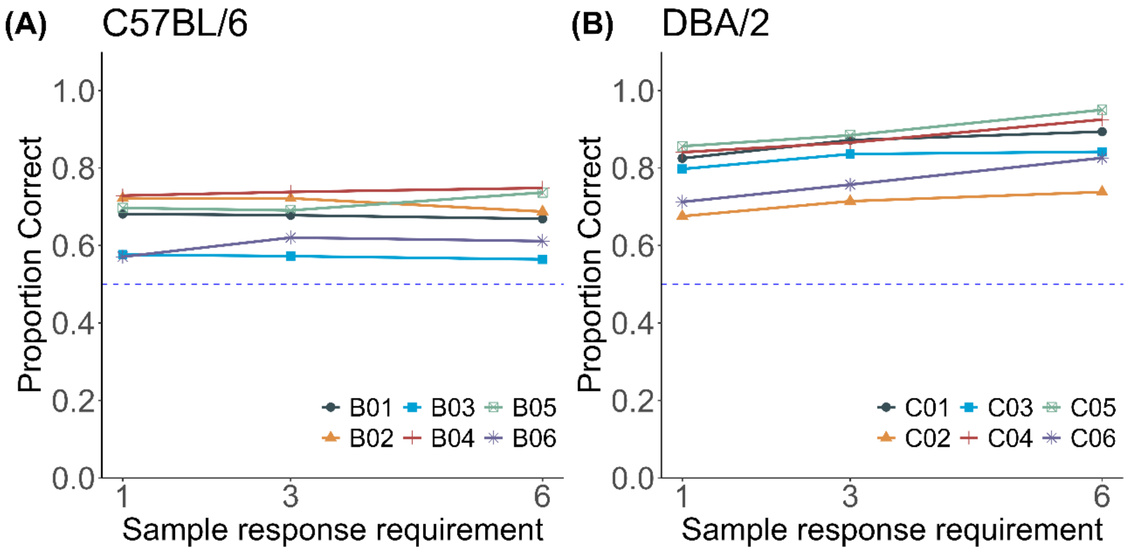

Fig 2S. Individual data from DMTP test with variable delay intervals. The horizontal axis represents the ratio of target to distractor luminance. Group data are shown in figure 3(C).

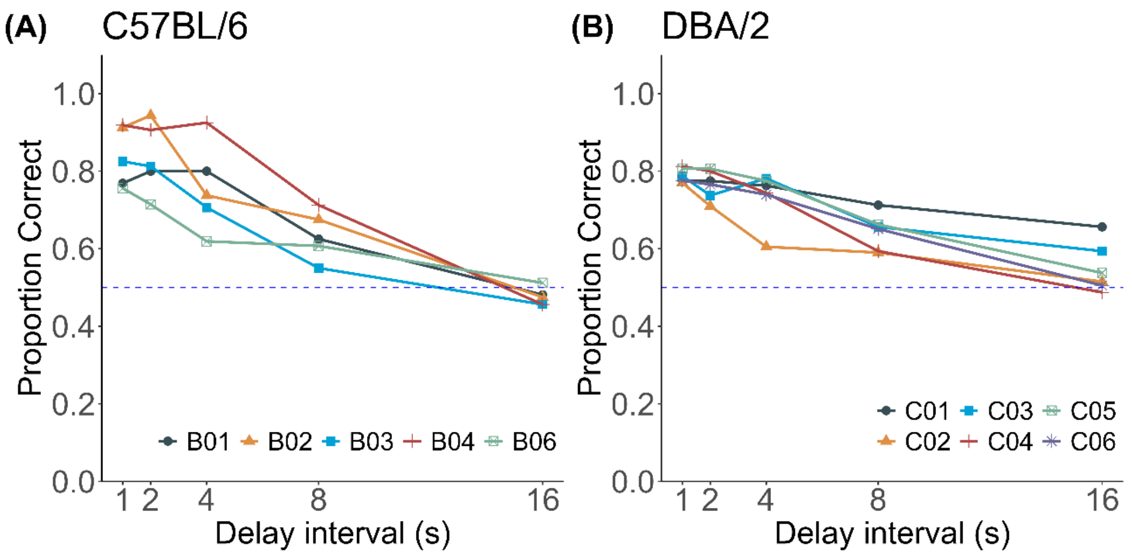

Fig3S. Individual data from DNMTP test with variable sample responses. The horizontal axis represents the ratio of target to distractor luminance. Group data are shown in figure 4(B).

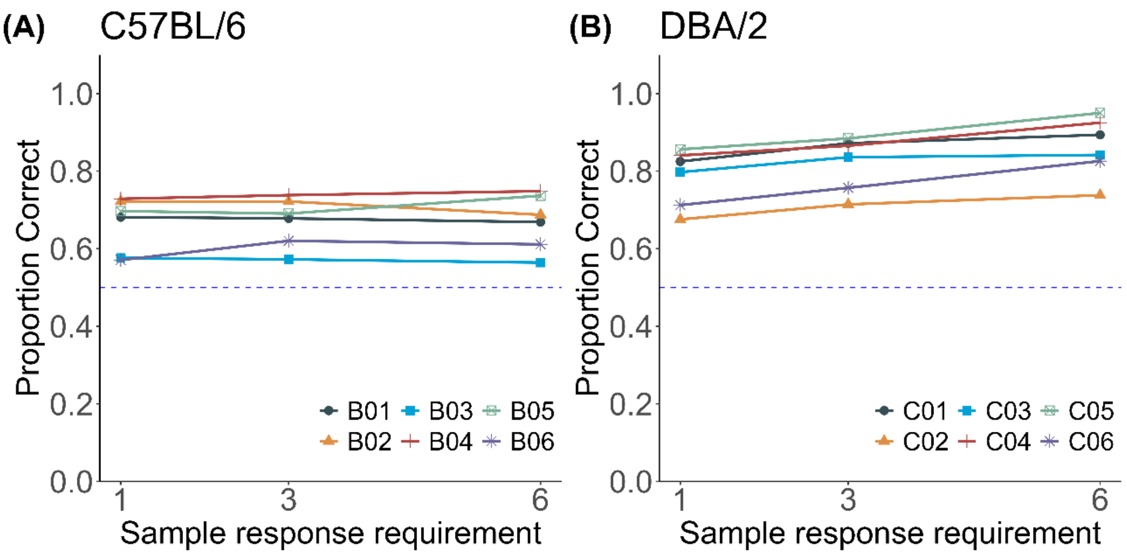

Fig4S. Individual data from DMTP test with variable sample responses. The horizontal axis represents the ratio of target to distractor luminance. Group data are shown in figure 4(C).

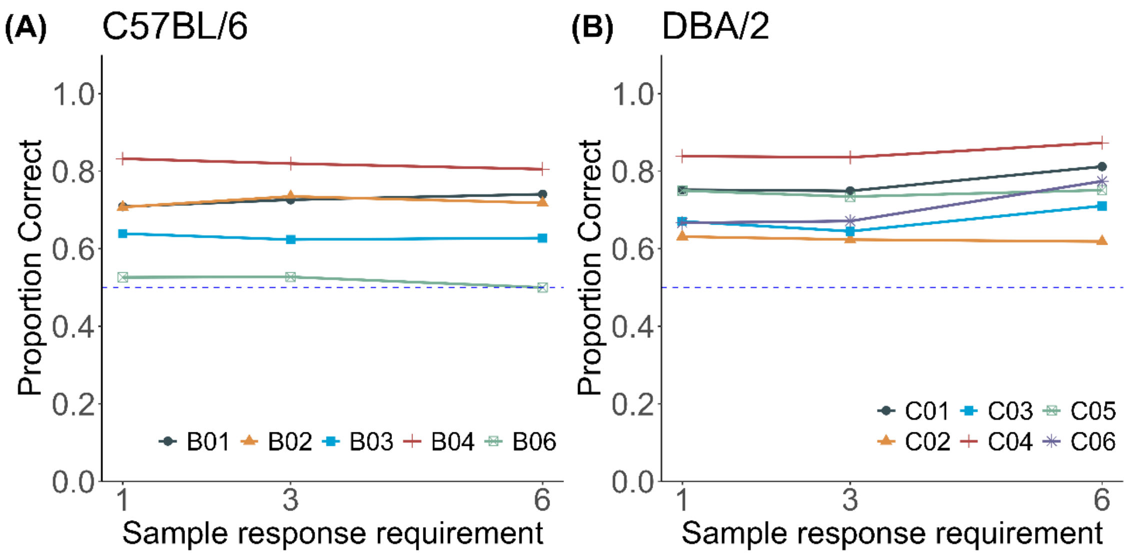

Supplement: Supplemental Information 2 [file peerj-13-19200-s002.pdf]
